# Lipoprotein Particle Predictors of Arterial Stiffness after 17 Years of Follow Up: The Malmö Diet and Cancer Study

**DOI:** 10.1155/2020/4219180

**Published:** 2020-04-28

**Authors:** Jacob Hartz, Ronald M. Krauss, Mikael Göttsater, Olle Melander, Peter Nilsson, Michele Mietus-Snyder

**Affiliations:** ^1^Department of Cardiology, Children's National Hospital, Washington D.C., USA; ^2^Boston Children's Hospital, Boston, MA, USA; ^3^University of California Benioff, Children's Hospital Oakland Research Institute, Oakland, CA, USA; ^4^Department of Clinical Sciences, Lund University, Skåne University Hospital, Malmö, Sweden; ^5^Clinical Research Center, Lund University, Skåne University Hospital, Malmö, Sweden; ^6^George Washington University School of Medicine and Health Sciences, Washington D.C., USA

## Abstract

**Background:**

Central arterial stiffness is a surrogate of cardiovascular risk and predicts cardiovascular mortality. Apolipoprotein B lipoproteins are also established cardiovascular risk factors. It is not known whether specific lipoprotein subclasses measured in the Malmö Diet and Cancer Study and previously shown to be associated with coronary heart disease also predict arterial stiffening after a mean period of 17 years.

**Methods:**

Lipoprotein particle analysis was performed on 2,505 men and women from Malmö, Sweden, from 1991 to 1994, and arterial stiffness was assessed by carotid-femoral pulse wave velocity (c-fPWV) on this same cohort from 2007 to 2012. Associations between c-fPWV and lipoprotein particles were determined with multiple linear regression, controlling for sex, presence of diabetes, waist-to-hip circumference, and smoking status at baseline, as well as heart rate (measured at the carotid artery), mean arterial pressure, antihypertensive and lipid-lowering medications, C-reactive protein (CRP), and age at the time of c-fPWV measurement.

**Results:**

The results confirm that triglycerides (TG) and high-density lipoprotein cholesterol (HDL-c) but not low-density lipoprotein cholesterol (LDL-c) predict c-fPWV. We identify a positive predictive association for very small, small, and medium (high risk), but not large LDL particles. There was a negative association for large HDL particles. The relationships between c-fPWV and high-risk LDL particles were unaffected by adjusting for LDL-c or CRP and were only mildly attenuated by adjusting for the homeostatic model for insulin resistance (HOMA-IR). Due to the collinearity of very small, small, and medium LDL particles and dyslipidemia (elevated TG and decreased HDL-c), the observed relationship between c-fPWV and high-risk LDL particles became insignificant after controlling for the concentration of HDL-c, large cholesterol-rich HDL particles, and TG.

**Conclusions:**

The development of central arterial stiffness previously associated with combined dyslipidemia may be mediated in part by LDL particles, particularly the very small-, small-, and medium-sized LDL particles.

## 1. Introduction

The infiltration of apolipoprotein B (apoB) containing lipoproteins within the vessel wall, release of inflammatory cholesterol crystals, and the development of fatty streaks are features of early atherosclerosis [1], but their influence on central arterial stiffening is not known. Many cardiovascular disease (CVD) risk factors associated with insulin resistance, (notably smoking [2], non-HDL-c [3], hypertension [4], and adiposity [5]), are also associated with apoB elevation [6]. There is a direct correlation between plasma apoB concentration and the number of atherogenic lipoprotein particles in circulation, 90-95% of which are LDL particles that can enter and be retained in the arterial wall [7, 8]. Further, increasing evidence links CVD events more closely to the size and concentration of the apoB lipoproteins than to their cholesterol content per se [9, 10].

A recent analysis using data from the Malmö Diet and Cancer Study found that hyperglycemia and insulin resistance, central adiposity, hypertriglyceridemia, and decreased HDL-c, but not elevated LDL-c, were associated with increased central arterial stiffness measured by carotid-femoral pulse wave velocity (c-fPWV) [11]. The c-fPWV measurement in meters/second represents the time required for the pulse to propagate from the carotid to the femoral artery. Central arterial stiffness is an independent risk factor for atherosclerosis [12, 13] and all-cause CVD morbidity and mortality [14–17]. The same combination of risk factors associated with c-fPWV in the Malmö cohort correlates with the presence of increased concentrations of small and total LDL particles across the lifespan. This relationship has been described in school-aged children [18] and in adults [19]. Previous longitudinal analyses within the Malmö cohort found that an atherogenic lipoprotein phenotype consisting of elevations in TG and apoB-containing lipoproteins, specifically increased concentrations of large very low-density lipoprotein (VLDL) particles and small and medium LDL particles, together with decreased HDL-c and decreased concentrations of large HDL particles, predicted increased risk of CVD events better than the traditional measure of LDL-c [20].

The aim of the study, therefore, was to determine if specific lipoprotein subclasses measured in the Malmö cohort have a stronger relationship with c-fPWV than LDL-c.

## 2. Materials and Methods

The Malmö Diet and Cancer Study has followed a robust cohort of adults from Malmö, Sweden, since 1991. The specifics of the study design and data collection have been detailed previously [11, 21]. In summary, the participants were originally enrolled from 1991 to 1994 with a follow-up period from May 2007 to September 2012. The original cohort included 6,103 individuals, and the follow-up included 3,734 participants from the original cohort. The study protocol conforms to the ethical guidelines of the 1975 Declaration of Helsinki and was approved by the ethical committee at Lund University, Lund, Sweden (baseline I.D. LU-5190, follow-up I.D. 532-2006). A written, informed consent was obtained from all participants.

### 2.1. Baseline Measurements

In the initial cohort from 1991 to 1994, the evaluation included a self-administered questionnaire, vital signs, anthropometric measures, and fasting laboratory samples. Sex, use of lipid-lowering medications, and smoking status were self-reported. The diagnosis of diabetes mellitus, either type 1 or type 2, was based on self-report, use of pharmaceutical treatment of diabetes, and/or a fasting blood glucose of at least 7.0 mmol/L (126 mg/dL). Vital signs and anthropometric measures included blood pressure, height, weight, and waist circumference. The fasting laboratory samples included C-reactive protein (CRP), serum glucose, glycosylated hemoglobin, insulin, total cholesterol, HDL-c, and TG, with calculated LDL-c. The homeostatic model of insulin resistance (HOMA-IR) was calculated by the product of fasting insulin (*μ*U/L) and glucose (mmol/L) divided by 22.5 [22]. The LDL-c was calculated using Friedewald's formula [23]. Lipoprotein particle subclasses were measured from 1991 to 1994 using ion mobility analysis [20], a technique that sensitively and directly quantifies lipoprotein particles as a function of particle diameter [24]. Both intra- and interassay CV's are <1.0% for LDL particles of all sizes. We created a new variable of “high-risk” LDL particles that included very small, small, and medium LDL particles, the subclasses with the largest positive association with arterial stiffness.

### 2.2. Follow-Up Measurements

The follow-up survey data used in these analyses included physical examination and noninvasive vascular testing that took place from May 2007 to September 2012. The use of blood pressure and lipid-lowering medications was self-reported. Hypertension was defined as a self-reported history of hypertension, ongoing blood pressure-lowering treatment, systolic blood pressure of at least 140 mmHg, or diastolic blood pressure of at least 90 mmHg at the time of the c-fPWV measurement, per established multisociety guidelines [25].

The c-fPWV was measured only at follow-up. The measurement was performed using applanation tonometry (SphygmoCor, AtCor Medical, Australia) with patients in supine position after five minutes of rest. The distance from the carotid to the femoral artery was measured using the “subtraction method” recommended by current vascular stiffness research guidelines to estimate the distance from the site of maximal carotid pulsation to maximal femoral pulsation (the suprasternal notch to the femoral recording site minus the suprasternal notch to the carotid recording site) [26]. With simultaneous electrocardiography registration, the difference between the time from the peak of the *R*-wave to the foot point of the pulse wave at the carotid and foot point of the femoral arteries is calculated and represents the time required for the pulse to travel the distance measured. The number of successful measurements in each individual varied from one to five, with a goal of three measurements (86.7% of cases). Results are based on mean c-fPWV from these assessments. The mean number of c-fPWV measurements obtained on each participant was three with a mean coefficient of variation of 6.3% (SD 4.4). This examination also included two blood pressure measurements (OMRON M5–1 IntelliSense) after five minutes of supine rest immediately before c-fPWV measurement. Mean arterial pressure was calculated as [(2 × diastolic pressure) + systolic pressure]/3.

### 2.3. Statistical Analysis

**Figure 1:**
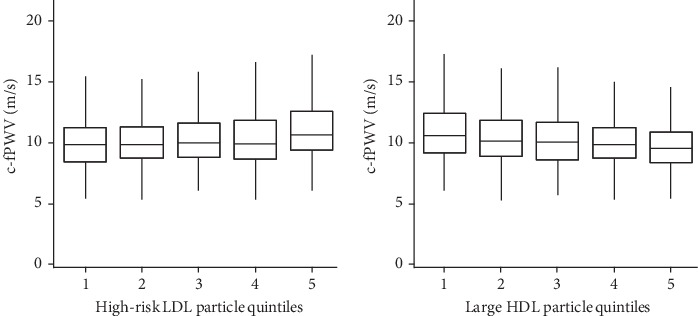
Associations of lipoprotein subspecies and arterial stiffness. Box-plot of c-fPWV by quintile of lipoprotein particles. High-risk LDL particles includes very small, small, and medium-sized LDL particles. Boxes contain 50% of observations, and bars depict the 95% confidence intervals. Horizontal lines indicate median value. c-fPWV: average carotid-femoral pulse wave velocity; HDL: high-density lipoprotein; LDL: low-density lipoproteins.

Analysis was conducted in R (R Core Team, 2014, version 3.4.3) and Figure 1 was produced using the R package ggplot2. Descriptive analyses of the study cohort were summarized, and results for males and females were compared using Student's *t*-test for parametric continuous variables, Mann–Whitney *U* tests for nonparametric continuous variables, and chi-squared tests for categorical data. To determine the predictive value of lipoprotein subfractions obtained at baseline and c-fPWV measured 17 years later, we performed multiple regression analysis. Model 1 controlled for sex, presence of diabetes, waist-to-hip circumference, smoking status at baseline, heart rate (measured from the carotid artery), mean arterial pressure, and whether or not participants were taking blood pressure-lowering medications and/or lipid-lowering medications at the time of c-fPWV measurement. Because of an expected significant positive correlation between age at the follow-up visit and the c-fPWV measured at that visit (*r* = 0.405, *p* < 0.001), age at the time of C-fPWV assessment is included in the adjusted regression model.

Model 2 included the variables in Model 1 and also controlled for CRP. Individuals with missing data were excluded from the analysis along with patients with a triglyceride level greater than 4.52 mmol/L (>400 mg/dL). In each analysis, the natural logarithms of c-fPWV, CRP, TG, and HDL-c and the individual lipoprotein particle fractions and particle ratios were used to achieve normal distributions. Total cholesterol and LDL-c values were normally distributed and not log transformed. Spearman rank correlation analyses of HOMA-IR and CRP, respectively, with all of the lipoprotein subspecies were considered. Interaction terms were created for HOMA-IR, CRP, and for sex with high-risk particles to further explore associations of these factors with arterial stiffness.

## 3. Results

A total of 2,485 participants had both lipoprotein particle and c-fPWV measurements. Sixty-two percent of the cohort was female with significant variation by sex across multiple traditional and lipid subspecies biomarkers. (Table 1) The mean age of the participants was 56 years at baseline, and mean follow-up time was 16.9 years. At follow-up, 54.4% of participants were taking a blood pressure-lowering medication and 41.9% were taking a lipid-lowering medication.

The results of the multiple regression analysis evaluating baseline lipid and lipoprotein particle predictors of c-f PWV at follow-up in a fully adjusted model are presented in Table 2. As reported previously in an overlapping but distinct Malmö cohort [11], TG (*β* = 0.028, *p* < 0.001) and HDL-c (*β* = −0.068, *p* < 0.001), but not LDL-c (*β* = 0.003, *p* = 0.46) were significant predictors of c-f PWV. Among LDL particle subfractions, there were statistically significant relationships with c-fPWV for very small- (*β* = 0.039, *p* = 0.02), small- (*β* = 0.039, *p* = 0.03), and medium-sized (*β* = 0.047, *p* = 0.01) but not large LDL particles (*β* = 0.009, *p* = 0.6), with a significant relationship for total LDL particles (*β* = 0.038, *p* = 0.03). There were no significant relationships between c-fPWV and particle concentrations of VLDL or large intermediate-density lipoprotein (IDL) subfractions, while there was an inverse association with small IDL particles (IDL-2, *β* = −0.042, *p* = 0.02). Finally, arterial stiffness was inversely associated with the concentration of large HDL particles (*β* = −0.068, *p* < 0.01), but not with concentrations of either small HDL particles or total HDL particles; the latter typically more abundantly populated with small HDL subspecies. Controlling for CRP (Table 2, Model 2) did not change these relationships.

When visualized incrementally, the augmentation of arterial stiffness is most evident at the highest three quintiles of high-risk LDL particles, whereas the inverse association of large HDL particles with c-fPWV is apparent across the full distribution (Figure 1).

The significant relationship between c-fPWV and high-risk very small, small, and medium LDL particles was unaffected by adjusting for LDL-c (*β* = 0.02, *p* = 0.03) but became insignificant after controlling for HDL-c (*β* = 0.01, *p* = 0.4), large HDL particles (*β* = 0.01, *p* = 0.4), and TG (*β* = 0.01, *p* = 0.6). Despite the strong inverse relationship between insulin resistance, as estimated by HOMA-IR, and lipoprotein species distribution (Table 3), associations between c-fPWV and high-risk LDL particles (*β* = 0.02, *p* = 0.046) and large HDL particles (*β* = −0.03, *p* < 0.001) remained significant after adjustment for HOMA-IR.

The relationships noted between all lipoprotein subspecies and sex, HOMA-IR, and inflammation were further probed. A significant interaction emerged between sex and high-risk particles but inclusion of this term did not change the final model that had already been adjusted for sex. Both HOMA-IR and CRP are strong correlates of virtually all lipoprotein subspecies but neither exhibited a significant interaction with any of the lipoprotein subclasses associated with c-fPWV, including high-risk particles (these interaction data not shown).

## 4. Discussion

In this study, we examined the association between baseline measurements of specific lipoprotein subclasses and c-fPWV after 17 years of follow-up of a middle-aged population from Malmö, Sweden. We found that levels of TG and very small-, small-, and medium-sized LDL particles, but not large LDL particles (subspecies defined by ES-DMA criteria [24]) nor LDL cholesterol, are predictors of subsequent increased arterial stiffness, while both HDL-c and the concentration of large HDL particles show an inverse predictive association. These findings reflect the cardiometabolic risk associated with insulin resistance, mediated in part through the atherogenic triad of elevated TG-rich lipoproteins (i.e., chylomicron remnants, large VLDL, VLDL remnants, and IDL), low HDL-c, and increased concentrations of very small-, small-, and medium-sized LDL particles. To our knowledge, this is the first demonstration of a longitudinal association between lipoprotein particles predicting later arterial stiffness, with differentiation of risk by the concentrations of LDL particle size subclasses.

There was an inverse association between vascular stiffness and the concentration of large cholesterol-enriched HDL particles and therefore with tightly correlated HDL-c concentration, consistent with a protective role for HDL that is incompletely understood [27]. Structural and functional properties of HDL particles may help explain their negative association with arterial stiffness [28]. Variation in the relative phospholipid and cholesterol content in large particles in a lean cohort as compared to persons with obesity or type 2 diabetes, has been linked to the inverse association of large HDL with c-fPWV [29]. Recent longitudinal analyses in men with CVD undergoing lifestyle modification therapy also suggest that HDL-c and the closely associated large HDL-P levels best predict ex vivo HDL cholesterol efflux capacity [30].

It is of interest that despite the association of plasma TG with c-fPWV, no significant associations were observed for particle concentrations of the major lipoprotein transporters of TG (VLDL and large IDL subclasses). This suggests that triglyceride enrichment of these precursor particles, rather than increases in their numbers per se, may be relevant to the development of arterial stiffness, possibly due to the resultant increased generation of small and medium LDL particles, both significantly associated with c-fPWV in this analysis. The weak inverse association of c-fPWV with smaller IDL particles may be a consequence of the direct correlations of this fraction with HDL-c and large HDL particles as reported previously in the Malmö cohort [20] and confirmed here (data not shown). CRP showed an inverse correlation with these same three lipoprotein subspecies (Table 3), but adjusting for CRP in Model 2 only minimally attenuated the inverse relationship between c-fPWV and small IDL.

### 4.1. Independent Role Insulin Resistance

Insulin resistance [31] is a known predictor of arterial stiffness. The association between c-fPWV and HOMA-IR was confirmed in this Malmo cohort (*r* = 0.1675, *p* < 0.001) and was also shown to be a strong correlate of most lipoprotein species (Table 3), but the relationship between insulin resistance and arterial stiffness appears to be independent of the role of lipoprotein particles. A distinct pathway for insulin resistance is consistent with the established role for the advanced glycation end products and nitric oxide dysregulation of insulin resistance and prediabetes in the pathogenesis of arterial stiffness that progresses with both type 1 and type 2 diabetes [32]. Additional factors implicated in lipid metabolism, but not measured in this cohort, may also be functional. For example, secretory phospholipase A2 activity, an independent CVD risk factor [33], is associated not only with smaller LDL particles but also with exposure of site A on the apoB lipoprotein that displays increased affinity for proteoglycan binding in the arterial wall [34, 35].

### 4.2. Mechanistic Insight to Vascular Stiffness

Arterial stiffness has become a reliable and reproducible noninvasive surrogate of cardiovascular risk, predicting cardiovascular mortality in hypertensive patients, independent of age [36]. Age has been the prevailing mechanistic explanation for vessel stiffening, attributed to changes in elastin, collagen, nitric oxide bioavailability, and advanced glycation end product crosslinking with or without calcification within the vessel wall matrix [26, 37]. As noted, many of these processes are related to insulin resistance [38] and inflammation [39]. The current report suggests that the previous associations between biomarkers of both processes, HOMA-IR and CRP, with vascular stiffening may be mediated in parallel with the pathophysiology of atherogenic dyslipoproteinemia.

Central arterial stiffness has previously been associated with coronary vessel atherosclerotic plaque formation [40]. The associations described here, in a large, well-characterized longitudinal cohort, add a plausible mechanistic association between the atherogenic infiltration of very small, small, and medium LDL particles into the central vascular wall and the development of arterial stiffness. This is consistent with research demonstrating increased carotid intima-media thickness and stiffening in obese, insulin-resistant, and diabetic youth [41]. Increased carotid intima-media thickness and stiffening are specifically associated with the accumulation of LDL particles in adults [10]. In addition, there is evidence in adults that hyperglycemia and the development of small LDL particles have an additive effect on the severity of arterial stiffening, assessed by brachial-ankle PWV [42]. Stiffer arterial vessels have increased permeability and are hypothesized to be more susceptible to LDL particle infiltration [43]. Yet, the importance of lipoproteins in the multifactorial pathogenesis of arterial stiffening remains incompletely defined, as recognized in the 2015 statement from the American Heart Association on vascular stiffening [26].

### 4.3. Limitations

Although this study has many strengths, including a long follow-up period and large cohort size, it is limited by the lack of c-fPWV measurements at baseline. Similarly, we can draw inferences, but no conclusions regarding causality in this association study. Although there was low attrition, the surviving participants with c-fPWV measurements may be healthier than the original study population. Furthermore, patients were excluded from c-fPWV measurement if they had cardiac arrhythmias. The generalizability of these findings will require further prospective study. Although we adjusted for medication use, the lack of detailed medication histories limits our ability to determine the role of specific blood pressure- and/or lipid-lowering medications that may have a range of effects on arterial stiffness [44, 45]. Finally, the findings of this longitudinal study from Southern Sweden may not be generalizable to populations with different racial, ethnic, and socioeconomic characteristics.

## 5. Conclusions

The development of central arterial stiffness may be mediated in part by LDL particles, particularly the very small-, small-, and medium-sized LDL particles (180.0–219.9 Å). The inverse association described between HDL-c and arterial stiffness may be attributed to large, cholesterol-rich HDL particles, although a putative protective role for HDL particles remains incompletely understood. These relationships between lipoprotein subspecies and vascular stiffness are consistent with their previously recognized associations with CVD. Taken together, these findings are hypothesis generating and warrant further evaluation. They underscore the importance of effective risk stratification and optimization of lipid metabolism at a younger age when vascular stiffening is already evident [29] and atherosclerosis is arguably most reversible [46].

## Figures and Tables

**Table 1:** Characteristics of study participants who underwent carotid-femoral pulse wave velocity measurement included in the regression analysis.

	Males	Females	*p* value
	*N* = 945	*N* = 1540	
Age at initial screening (years)	56.1 (5.8)	56 (5.5)	0.7^∗^
Age at c-fPWV (years)	72.8 (5.7)	72.9 (5.4)	0.5^∗^
Follow-up time (years)	16.7 (1.5)	16.9 (1.5)	<0.001^∗^
BMI (kg/m^2^)	25.8 (3.1)	25.1 (3.8)	<0.001^∗^
Waist-to-hip ratio	0.9 (0.1)	0.8 (0.1)	<0.001^∗^
Smoker, current	163 (18%)	256 (17%)	<0.001^∗∗^
Smoker, occasional	48 (5%)	67 (4%)	<0.001^∗∗^
Smoker, former	418 (45%)	443 (29%)	<0.001^∗∗^
Smoker, never	294 (32%)	749 (49%)	<0.001^∗∗^
Diabetes diagnosis	71 (8%)	59 (4%)	<0.001^∗∗^
Lipid-lowering medication at follow up	323 (34%)	412 (27%)	<0.001^∗∗^
Antihypertensive medication at follow up	530 (56%)	823 (53%)	0.2^∗∗^
HBA_1C_ (%)	4.8 (4.5, 5)	4.8 (4.5, 5)	0.512
Glucose, fasting (mmol/L)	5 (4.7, 5.3)	4.8 (4.5, 5.1)	<0.001
Insulin level (mmol/L)	7 (5, 9)	6 (4, 8)	<0.001
HOMA-IR	1.5 (1, 2.1)	1.2 (0.8, 1.8)	<0.001
C-reactive protein (nmol/L)	1.2 (0.6, 2.3)	1.1 (0.6, 2.4)	0.8
Total cholesterol (mmol/L)	6 (1)	6.2 (0.1)	<0.001^∗^
Triglycerides (mmol/L)	1.3 (0.9, 1.7)	1 (0.8, 1.4)	<0.001
HDL-c (mmol/L)	1.2 (1, 1.4)	1.5 (1.3, 1.8)	<0.001
LDL-c (mmol/L)	4.1 (0.9)	4.1 (1)	0.77^∗^
TG : HDL-c ratio (ideal <0.9 in mmol/L)	1.1 (0.7, 1.6)	0.7 (0.5, 1)	<0.001
Large VLDL-P (mmol/L)	9 (6, 12)	8 (5, 11)	<0.001
Medium VLDL-P (mmol/L)	34 (27, 44)	32 (23, 43)	<0.001
Small VLDL-P (mmol/L)	48 (39, 60)	51 (40, 66)	<0.001
Large IDL-P(mmol/L)	112 (91, 140)	109 (86, 139)	0.03
Small IDL-P (mmol/L)	158 (120, 223)	225 (166, 298)	<0.001
Large LDL-P(mmol/L)	433 (331, 542)	392 (301, 506)	<0.001
Medium LDL-P (mmol/L)	121 (81, 204)	80 (61, 112)	<0.001
Small LDL-P(mmol/L)	70 (48, 114)	52 (39, 70)	<0.001
Very small LDL-P (mmol/L)	104 (812, 136)	88 (69, 113)	<0.001
Total LDL-P (mmol/L)	788 (624, 966)	623 (498, 812)	<0.001
Large HDL-P (nmol/L)	984 (690, 1446)	1801 (1211, 2568)	<0.001
Small HDL-P (nmol/L)	2567 (1896, 3391)	2706 (2078, 3593)	<0.001
Total HDL-P (nmol/L)	3682 (2829, 4760)	4663 (3669, 5859)	<0.001
c-fPWV (m/s)	10.4 (8.9, 12.2)	9.9 (9, 11)	<0.001

For continuous variables, normally distributed characteristics are expressed with mean (SD) and skewed data with median (interquartile range). Categorical data are expressed as percentages. ^∗^Comparisons of parametric data are made using Student's *t*-test. ^∗∗^Comparisons of categorical data are made using chi square tests. All other comparisons of mean ranks for nonparametric data use the Mann–Whitney *U* test. Abbreviations: c-fPWV: carotid-femoral pulse wave velocity; HDL-c: high-density lipoprotein cholesterol; HDL-P: high-density lipoprotein particles; HOMA-IR: homeostatic model assessment of insulin resistance; IDL-P: intermediate-density lipoprotein particles; LDL-c: low-density lipoprotein cholesterol; LDL-P: low-density lipoprotein particles; VLDL-P: very low-density lipoprotein particles.

**Table 2:** Multiple linear regression analysis for baseline and follow-up determinants of the dependent variable, ln carotid–femoral pulse wave velocity.

	Model 1		Model 2	
	Beta	*p* value	Beta	*p* value
Cholesterol (total)	0.003	0.86	0.010	0.6
Triglycerides	0.071	<0.01	0.070	<0.01
HDL-c	-0.080	<0.01	-0.070	<0.01
LDL-c	0.011	0.52	0.020	0.4
Large VLDL-P	0.004	0.83	0.002	0.9
Medium VLDL-P	-0.001	0.94	-0.002	0.9
Small VLDL-P	-0.011	0.53	-0.011	0.5
Large IDL-P	0.010	0.54	0.013	0.5
Small IDL-P	-0.042	0.02	-0.037	0.03
Large LDL-P	0.009	0.60	0.009	0.6
Medium LDL-P	0.047	0.01	0.045	0.01
Small LDL-P	0.039	0.03	0.038	0.03
Very small LDL-P	0.039	0.02	0.040	0.02
Total LDL-P	0.038	0.03	0.037	0.04
Large HDL-P	-0.068	<0.01	-0.062	<0.01
Small HDL-P	0.002	0.92	-0.002	0.92
Total HDL-P	-0.026	0.13	-0.026	0.13
High-risk LDL-P^∗^	0.049	0.01	0.047	0.01

^∗^High-risk LDL-P contains very small, small, and medium LDL particles. Model 1 controlled for heart rate (measured from the carotid artery), mean arterial pressure at the time of the c-fPWV measurements, age at time of c-fPWV measurement, gender, fasting glucose ≥ 7 mmol, waist-to-hip circumference at baseline, smoking status at baseline, and whether participants were taking blood pressure-lowering medications and/or lipid-lowering medications at follow-up. Model 2 included the variables in Model 1 plus natural log transformed C-reactive protein. Abbreviations: HDL-c: high-density lipoprotein cholesterol; HDL-P: high-density lipoprotein particles; IDL-P: intermediate-density lipoprotein particles; LDL-c: low-density lipoprotein cholesterol; LDL-P: low-density lipoprotein particles; VLDL-P: very low-density lipoprotein particles.

**Table 3:** Correlation between HOMA-IR and C-reactive protein with lipoprotein particles.

	HOMA-IR	*p* value	C-reactive protein	*p* value
Large VLDL-P	0.28	<0.0001	0.0858	<0.001
Medium VLDL-P	0.21	<0.0001	0.0814	0.001
Small VLDL-P	0.07	0.0005	0.0446	0.03
Large IDL-P	-0.18	<0.0001	0.0680	<0.001
Small IDL-P	0.17	0.0005	-0.0624	0.001
Large LDL-P	0.09	<0.001	0.1126	<0.001
Medium LDL-P	0.32	<0.001	0.1310	<0.001
Small LDL-P	0.30	<0.001	0.1099	<0.001
Very small LDL-P	0.19	<0.001	0.0309	0.13
Total LDL-P	0.26	<0.001	0.1222	<0.001
Large HDL-P	-0.33	<0.001	-0.1781	<0.001
Small HDL-P	0.02	0.25	0.0358	0.08
Total HDL-P	-0.15	<0.001	-0.0787	<0.001
High risk LDL-P^∗^	0.28	<0.0001	0.0973	<0.001

^∗^High-risk LDL-P contains very small, small, and medium LDL particles. Abbreviations: HDL: high-density lipoprotein; HDL-P: high-density lipoprotein particles; HOMA-IR: homeostatic model assessment of insulin resistance; IDL-P: intermediate-density lipoprotein particles; LDL: low-density lipoprotein; LDL-P: low-density lipoprotein particles; VLDL-P: very low-density lipoprotein particles.

## Data Availability

The Malmö Diet Cancer data set is not in the public domain but the dataset analyzed for the current study is available from the Steering Committee of the Malmö Cohorts at Lund University (chair: Olle Melander) upon reasonable request (contact information above and also found at: http://epic.iarc.fr/centers/sweden.php).

## Abbreviations

apoB: Apolipoprotein B
c-fPWV: Carotid-femoral pulse wave velocity
CRP: C-reactive protein
CVD: Cardiovascular disease
ES-DMA: Electrospray differential mobility analysis
HDL-c: High-density lipoprotein cholesterol
HOMA-IR: Homeostatic model of insulin resistance
IDL: Intermediate-density lipoproteins
LDL-c: Low-density lipoprotein cholesterol
TG: Triglycerides
VLDL: Very low-density lipoproteins.
